# Supplementary material for: Structure of the Arabidopsis guard cell anion channel SLAC1 suggests activation mechanism by phosphorylation
Source: Nat Commun. 2022 May 6;13:2511. doi: 10.1038/s41467-022-30253-3 (PMC9076830; doi:10.1038/s41467-022-30253-3)
Supplement: Supplementary file 1 — Supplementary Information [file 41467_2022_30253_MOESM1_ESM.pdf]

Supplementary Information

Structure of the *Arabidopsis* guard cell anion channel SLAC1 suggests activation mechanism by phosphorylation

Yawen Li[1,5], Yinan Ding[1,2,5], Lili Qu[1,2], Xinru Li[1], Qinxuan Lai[1], Pingxia Zhao[3], Yongxiang Gao[4], Chengbin Xiang[3], Chunlei Cang[1,2,6], Xin Liu[1,6] & Linfeng Sun[1,6]

This file includes:
Supplementary Figs. 1 to 7
Supplementary Tables 1 to 2

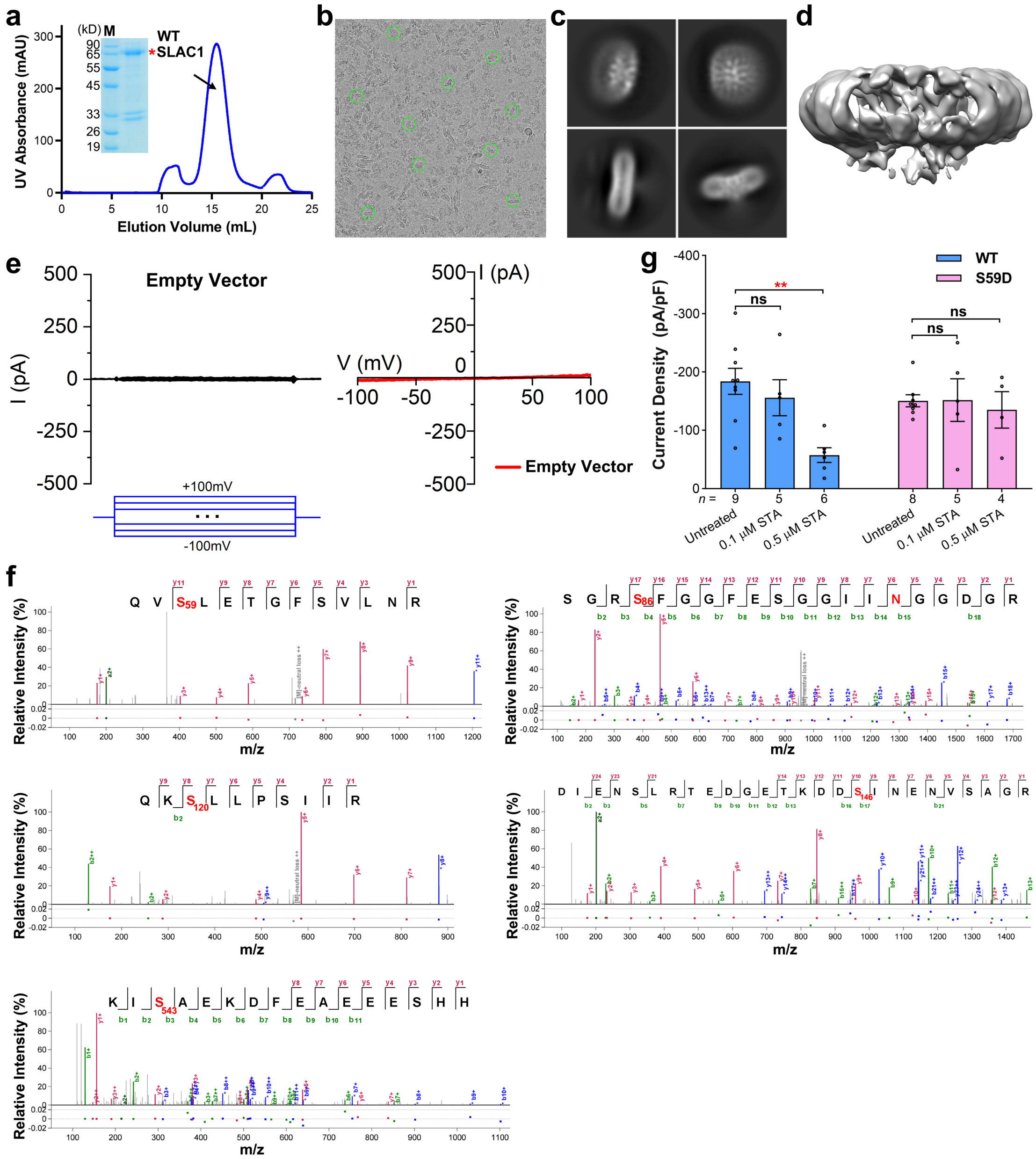

Supplementary Fig. 1. Characterizations of the wild type *At*SLAC1 protein in HEK293 cells.

a, A representative gel filtration analysis of the WT *At*SLAC1. The mono-disperse peak of the protein indicated by the arrow and verified by Coomassie-blue-staining SDS-PAGE (inner panel), suggests a good solution behavior of the sample. Experiments have been repeated for five times with similar results. **b,** A cryo-EM image of the WT *At*SLAC1. Representative particles are indicated by green circles. **c,**

Typical 2D classification images of the WT *At*SLAC1. **d,** 3D reconstruction map of the WT *At*SLAC1. **e,** Representative current traces recorded using a step protocol (-100 mV to +100 mV, 20 mV step; $V_{holding}$ = 0 mV) or a ramp protocol (-100 mV to +100 mV in 1 s; $V_{holding}$ = 0 mV) in HEK293T cells expressing the empty pCAG vector as a negative control. **f,** Mass spectrometry analysis of the WT *At*SLAC1 sample purified from HEK293F cells. Five serine residues, coloured red, were identified to be phosphorylated. **g,** Inward current densities for the WT AtSLAC1 and phosphomimetic mutant S59D at -100 mV in the untreated HEK293T cells, or cells pretreated with 0.1 μM or 0.5 μM staurosporine (STA) for 1 h, respectively. Independent experiments were repeated and the number of cells used (*n*) were indicated under each column. Significances were determined using one-way ANOVA with Dunnett's multiple comparisons test. ** P = 0.0016, ns = not significant. Data are represented as mean ± S.E.M.

a **Wild Type *At*SLAC1**

MERKQSNAHSTFADINEVEDEAEQELQQQENNNNKRFSGNRGPNRGKQRPFRGFSRQVS$_{59}$LETGF
SVLNRESRERDDKKSLPRSGRS$_{86}$FGGFESGGIINGGDGRKTDFSMFRTKSTLSKQKS$_{120}$LLPSIIRERDIE
NSLRTEDGETKDDS$_{146}$INENVSAGRYFAALRGPELDEVKDNEDILLPKEEQWPFLLRFPIGCFGICLGLSS
QAVLWLALAKSPATNFLHITPLINLVVWLFSLVVLVSVSFTYILKCIFYFEAVKREYFHPVRVNFFFAPWV
VCMFLAISVPPMFSPNRKYLHPAIWCVFMGPYFFLELKIYGQWLSGGKRRLCKVANPSSHLSVVGNFV
GAILASKVGWDEVAKFLWAVGFAHYLVVFVTLYQRLPTSEALPKELHPVYSMFIAAPSAASIAWNTIY
GQFDGCSRTCFFIALFLYISLVARINFFTGFKFSVAWWSYTFPMTTASVATIKYAEAVPGYPSRALALTLS
FISTAMVCVLFVSTLLHAFVWQTLFPNDLAIAITKRKLTREKKPFKRAYDLKRWTKQALAKKIS$_{543}$AEKDF
EAEEESHH

b **S59A *At*SLAC1**

MERKQSNAHSTFADINEVEDEAEQELQQQENNNNKRFSGNRGPNRGKQRPFRGFSRQVA$_{59}$LETGF
SVLNRESRERDDKKSLPRSGRS$_{86}$FGGFESGGIINGGDGRKTDFSMFRTKSTLSKQKS$_{120}$LLPSIIRERDIE
NSLRTEDGETKDDS$_{146}$INENVSAGRYFAALRGPELDEVKDNEDILLPKEEQWPFLLRFPIGCFGICLGLSS
QAVLWLALAKSPATNFLHITPLINLVVWLFSLVVLVSVSFTYILKCIFYFEAVKREYFHPVRVNFFFAPWV
VCMFLAISVPPMFSPNRKYLHPAIWCVFMGPYFFLELKIYGQWLSGGKRRLCKVANPSSHLSVVGNFV
GAILASKVGWDEVAKFLWAVGFAHYLVVFVTLYQRLPTSEALPKELHPVYSMFIAAPSAASIAWNTIY
GQFDGCSRTCFFIALFLYISLVARINFFTGFKFSVAWWSYTFPMTTASVATIKYAEAVPGYPSRALALTLS
FISTAMVCVLFVSTLLHAFVWQTLFPNDLAIAITKRKLTREKKPFKRAYDLKRWTKQALAKKIS$_{543}$AEKDF
EAEEESHH

Supplementary Fig. 2. Summary of the mass spectrometry results for the wild type *At*SLAC1 and S59A mutant.

a, Mass spectrometry result for the wild type *At*SLAC1. Residues assigned in the peptides are highlighted yellow and the identified phosphorylation site are coloured red. Five sites, S59, S86, S120, S146 and S543 are phosphorylated. **b,** Mass spectrometry result for the *At*SLAC1 S59A mutant. Residues assigned in the peptides are highlighted yellow and the identified phosphorylation site are coloured red. S146 and S543 are phosphorylated.

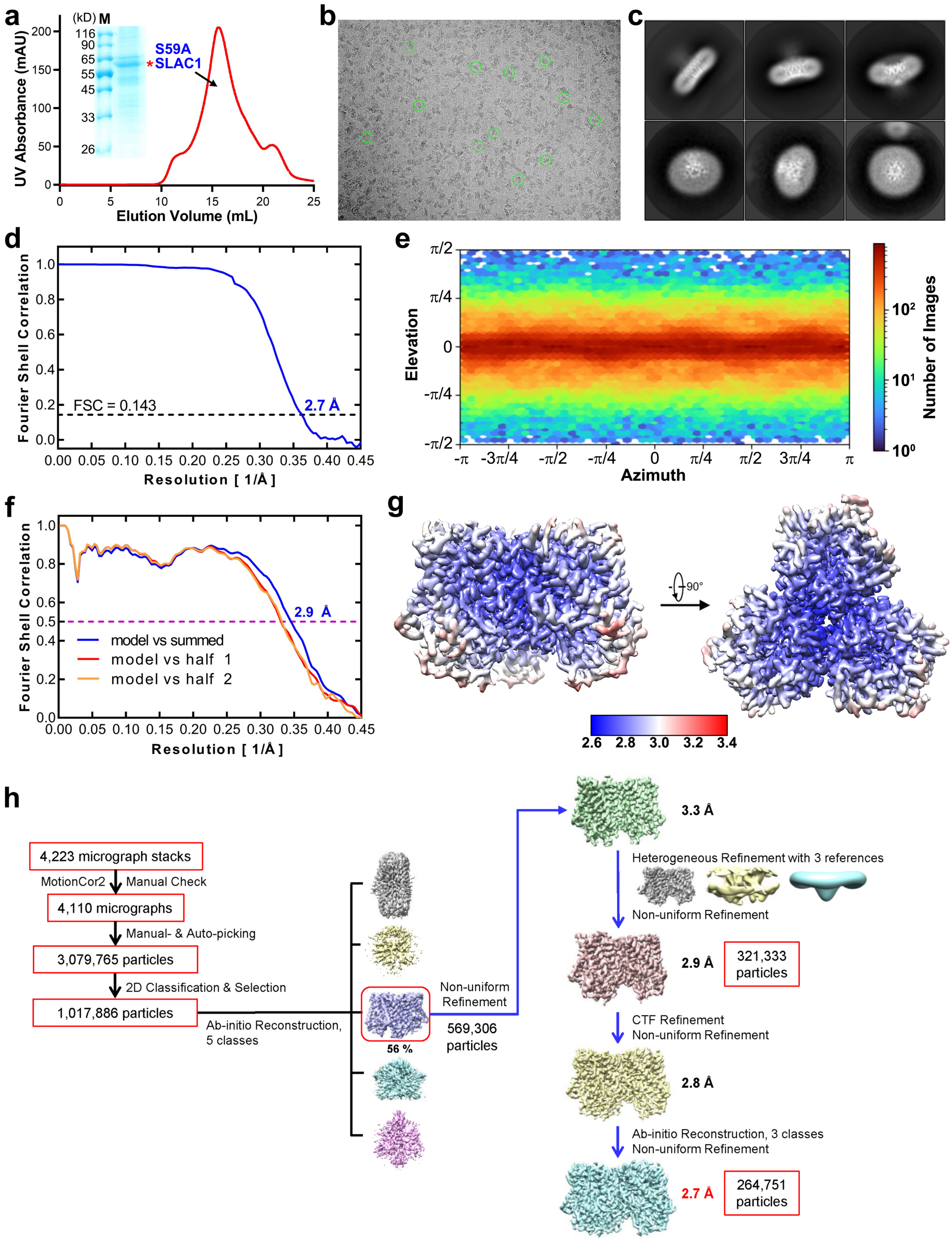

Supplementary Fig. 3. Structure determination for the *At*SLAC1 S59A mutant.

a, A representative gel filtration analysis of the *At*SLAC1 S59A mutant. Experiments have been repeated for five times with similar results. **b,** A representative cryo-EM image of the *At*SLAC1 S59A mutant. Particles are indicated by green circles. **c,** 2D classification images. **d,** The gold-standard Fourier shell correlation curve for the

overall map. The resolution reaches 2.7 Å by a threshold of 0.143. **e,f,g,** Euler angle distribution, local resolution map and FSC model curves of the *At*SLAC1 S59A map. FSC curves of the refined model versus the overall map of state-1 that it was refined against (blue); of the model refined in the first of the two independent maps used for the gold-standard FSC versus that same map (red); and of the model refined in the first of the two independent maps versus the second independent map (orange). The small difference between the FSC curve of the model refined in the first of the two independent maps used for the gold-standard FSC versus that same map (red) and that of the model refined in the first of the two independent maps versus the second independent map (orange) indicates that the refinement of the atomic coordinates did not suffer from overfitting. **h,** Flowchart for cryo-EM data processing. Details can be found in the "Image processing" session in Methods.

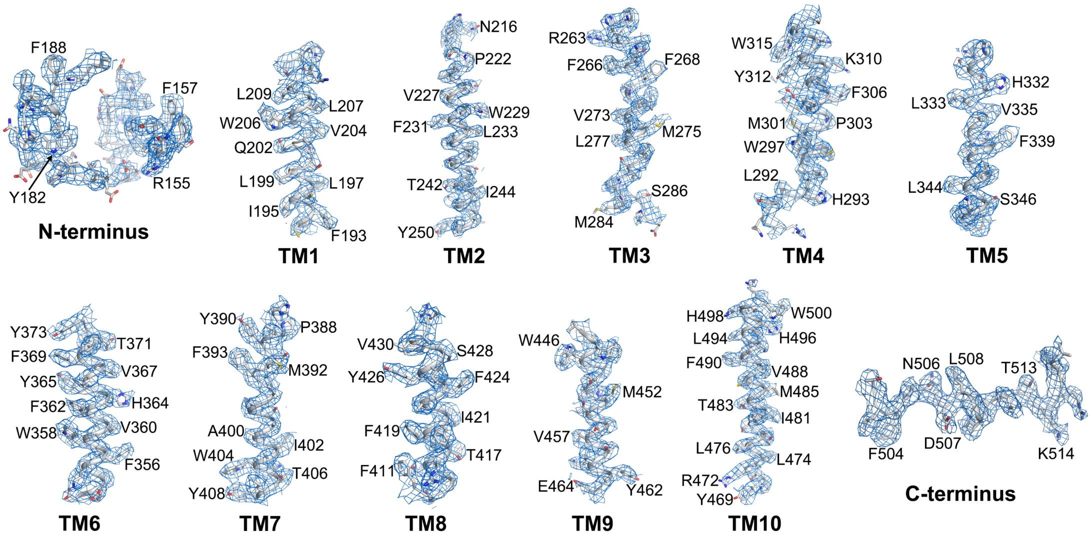

Supplementary Fig. 4. Representative EM densities for the *At*SLAC1 S59A mutant.

EM densities for the N-terminus, ten transmembrane helices, and the C-terminus are shown in marine blue mesh. Residues with notable side chains are labeled aside.

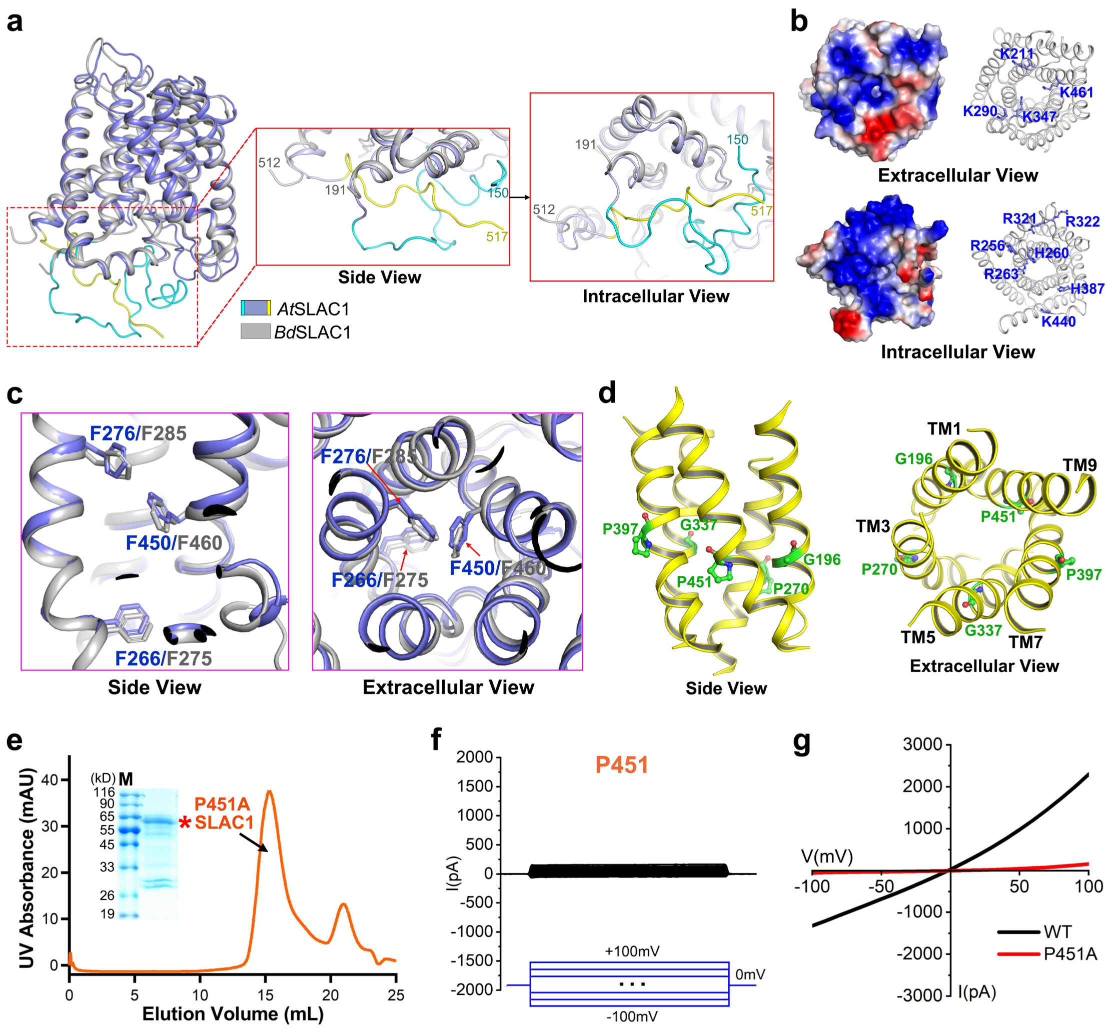

Supplementary Fig. 5. Characterizations of the pore-forming residues of *At*SLAC1.

a, Structure alignments between *At*SLAC1 and *Bd*SLAC1 (PDB code: 7en0). *Bd*SLAC1 is shown in light grey. The N-terminus, transmembrane domain and C-terminus of *At*SLAC1 are coloured cyan, slate blue and yellow, respectively. Two zoom-in views are shown for the extra N-terminus and C-terminus firstly observed in the *At*SLAC1 structure. The starting residue number of the N-terminus and the ending residue number of the C-terminus in the structures are labelled for each protein. **b,** The extracellular and intracellular surfaces of the pore are enriched in positively

charged residues. Two views of the electrostatic potential surface are shown for the transmembrane domain alone. The lysine or arginine residues are shown in sticks. **c,** The three pore-lining phenylalanine residues (shown in sticks) have conserved configurations in the *Bd*SLAC1 (coloured grey) and *At*SLAC1 (coloured slate blue) structures. Two perpendicular views are shown here. **d,** Kink residues in the pore-forming transmembrane helices. **e,** A representative gel filtration analysis of the *At*SLAC1 P451A mutant. Experiments have been repeated for three times with similar results. **f, g,** Representative voltage-clamp recording results for the P451A mutant.

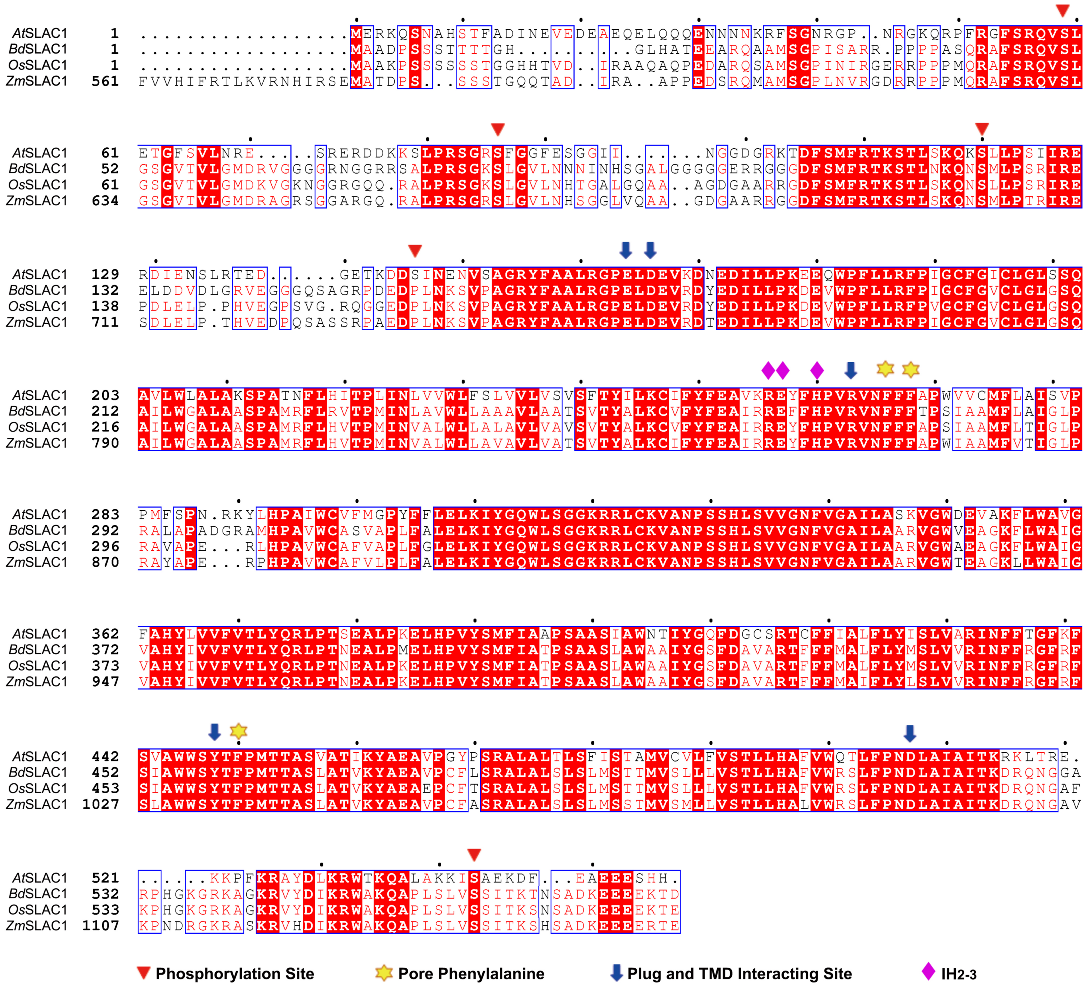

Supplementary Fig. 6. Sequence alignments of SLAC1 channel from *Arabidopsis thaliana* (*At*SLAC1), *Barachypodium distachyon* (*Bd*SLAC1), *Oryza sativa subsp. japonica* (rice, *Os*SLAC1) and *Zea mays* (maize, *Zm*SLAC1).

Sequences are aligned using ClustalW. The identified phosphorylation sites in the wild type *At*SLAC1, three pore-forming phenylalanine residues, key residues in $IH_{2\text{-}3}$, and the interacting sites between the plug and the transmembrane domain are indicated by different symbols.

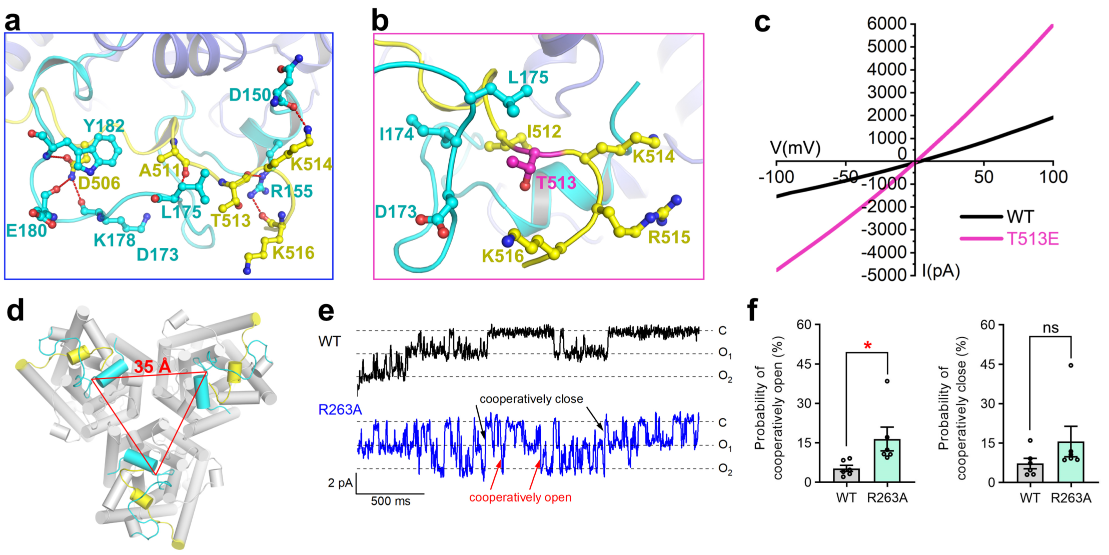

Supplementary Fig. 7. T513E increases the anion currents of *At*SLAC1 in HEK293 cells.

a, Zoomed view of the interactions between the N-terminus and C-terminus. Carbon atoms of the N-terminus is coloured cyan and those of the C-terminus are coloured yellow. **b,** Zoomed view of the T513 residue in the C-terminus. **c,** Representative current traces recorded using a ramp protocol (-100 mV to +100 mV in 1 s; $V_{holding}$ = 0 mV) for the WT *At*SLAC1 and T513E mutant. **d,** Locations of the plug structures in the *At*SLAC1 trimer. The N-terminus and C-terminus forming the plug are coloured cyan and yellow, respectively. The distance between two adjacent pores is about 35 Å as indicated by red lines. **e,** Single-channel currents recorded at -100 mV using cell-attached patch-clamp in HEK293T cells expressing WT *At*SLAC1 or R263A mutant. Dashed lines indicate different states of channels: close (C), one channel open (O_1) and two channels open (O_2). **f**, Probability of cooperatively open and cooperatively close of WT *At*SLAC1 or R263A mutant in single channel recordings. Independent experiments were repeated for each construct (WT, *n* = 6 cells; R263A, *n*

= 6 cells). Significances were determined using unpaired two-tailed *t*-test. *P = 0.0383, ns = not significant. Data are represented as mean ± S.E.M.

Supplementary Table 1. Statistics of cryo-EM data collection, processing, model refinement and validation

**Data Collection**	
EM equipment	FEI Titan Krios
Voltage (kV)	300
Detector	GATAN BioQuantum K3
Magnification	81000 x
Pixel size (Å)	1.1
Electron dose (e^-/Å^2)	50
Defocus range (μm)	-1.0 ~ -2.0
**Data Processing**	
Softwares	RELION 3.1, cryoSPARC v.3.2.0
Initial Number of Particles	3,079,765
Final Number of Particles	264,751
Symmetry	C3
Map Resolution (Å)	2.7
FSC Threshold	0.143
**Model Refinement**	
Map Sharpening B-factor (Å^2)	-140.7
Model Resolution (Å)	2.9
FSC Threshold	0.5
Protein residues	368
CC mask	0.86
**Validation**	
R.m.s. Deviations	
Bond lengths (Å)	0.007
Bond angles (°)	0.974
MolProbity Score	1.10
All-atom Clashscore	1.41
Rotamer Outliers (%)	0
Ramachandran plot	
Favored (%)	96.54
Allowed (%)	3.46
Outliers (%)	0

Supplementary Table 2. Time constant (tau) of channel open and close in single channel recordings

*At*SLAC1	Tau of open (ms)		Tau of close (ms)	
	One channel	Two channels	One channel	Two channels
**WT**	9.60 ± 2.35	7.50 ± 1.09	23.68 ± 6.21	7.13 ±3.19
**R263A**	12.83 ± 2.02	9.67 ± 2.87	22.02 ± 5.15	8.49 ±1.91
